# Preventive Gastroprotective Effect of a Functional Food Based on Quinoa (*Chenopodium quinoa* Willd.) and Quercetin in a Murine Model of Ibuprofen-Induced Gastric Damage

**DOI:** 10.3390/antiox14070893

**Published:** 2025-07-21

**Authors:** Maribel Valenzuela-González, José Luis Cárdenas-López, Armando Burgos-Hernández, Norma Julieta Salazar-López, Manuel Viuda-Martos, Mónica A. Villegas-Ochoa, Gustavo Martínez-Coronilla, J. Abraham Domínguez-Avila, Shela Gorinstein, Gustavo A. González-Aguilar, Rosario Maribel Robles-Sánchez

**Affiliations:** 1 Departamento de Investigación y Posgrado en Alimentos, Universidad de Sonora, Blvd. Luis Encinas y Rosales Col. Centro. A.P., Hermosillo 83000, Sonora, Mexico; maribelvalenzuelag4@gmail.com (M.V.-G.); joseluis.cardenas@unison.mx (J.L.C.-L.); armando.burgos@unison.mx (A.B.-H.); 2 Facultad de Medicina de Mexicali, Universidad Autónoma de Baja California, Dr. Humberto Torres Sanginés S/N, Centro Cívico, Mexicali 21000, Baja California, Mexico; norma.salazar@uabc.edu.mx (N.J.S.-L.); gustavoj@uabc.edu.mx (G.M.-C.); 3 IPOA Research Group, Centro de Investigación e Innovación Agroalimentaria y Agroambiental, Universidad Miguel Hernández (CIAGRO-UMH), Ctra. Beniel Km 3.1, 03312 Orihuela, Spain; mviuda@umh.es; 4 Centro de Investigación en Alimentación y Desarrollo, A.C., Carretera Gustavo Enrique Astiazarán Rosas, No. 46, Colonia La Victoria, Hermosillo 83304, Sonora, Mexico; mvillegas@ciad.mx (M.A.V.-O.); gustavo@ciad.mx (G.A.G.-A.); 5 SECIHTI-Centro de Investigación en Alimentación y Desarrollo, A.C., Carretera Gustavo Enrique Astiazarán Rosas, No. 46, Colonia La Victoria, Hermosillo 83304, Sonora, Mexico; abrahamdominguez9@gmail.com; 6 Department of Medicinal Chemistry and Natural Products, School of Pharmacy, The Hebrew University, Hadassah Medical School, Jerusalem 91120, Israel; shela.gorin@mail.huji.ac.il

**Keywords:** oxidative stress, gastric lesion, pseudocereals, gastroprotection, flavonoids

## Abstract

Nonsteroidal anti-inflammatory drug-based therapies are the cause of 20–30% cases of gastric lesions in chronic users worldwide. Co-medication with omeprazole (OMP) is the most commonly used option to prevent these lesions, although this carries risks of its own; thus, alternatives are being explored, such as dietary antioxidant therapies. The objective of this study was to evaluate the gastroprotective activity of quinoa (*Chenopodium quinoa* Willd.) on ibuprofen (IBP)-induced gastric ulcers in a rat model. Quinoa cookies were formulated with heat-treated quinoa using microwave radiation. The intestinal bioaccessibility of phenols and flavonoids, and the antioxidant activity of microwaved quinoa cookies (MQCs) were notably higher than quinoa cookies without thermal treatment (RQCs): 132% TPC, 52% TFC, 1564% TEAC vs. 67% TPC, 24% TFC, and 958% TEAC, respectively. Basal diets were supplemented with MQCs (20%) and quercetin (Q, 0.20%) as a reference flavonoid and administered for 30 days. Gastric lesions were induced by intragastric IBP doses, with OMP treatment as a positive control. Gastric damage index (macroscopic study), histological score (microscopic study), and plasma antioxidant enzyme activity (SOD and CAT) were evaluated. Macroscopic results showed that the addition of MQCs, Q, and OMP decreased the gastric damage index (GDI) by 50%, 40%, and 3%, respectively, as compared to IBP (GDI 100%). Histological analyses showed neutrophil infiltration and congested blood vessels in IBP-treated tissues; in contrast, the experimental diet groups showed lower infiltration for MQC > OMP > Q, respectively. A significant increase in SOD and CAT enzyme activity was observed in the MQC and Q groups as compared to the IBP group. We conclude that a reduction in the GDI and histological score was observed in IBP-induced murine models fed diets containing 20% MQC and 0.20% Q, demonstrating a preventive gastroprotective effect.

## 1. Introduction

The gastric mucosa is essential for various physiological functions, such as the protection that it provides against different gastrointestinal diseases that develop in this tissue, including gastrointestinal inflammation, peptic ulcer, gastrointestinal cancer, among others [1]. Gastric lesions are caused by an imbalance between the aggressive and defensive factors to which the gastric mucosa is exposed; when the defensive agents are overwhelmed, and therefore unable to provide sufficient protection, damage is generated by histopathological alterations [2]. One of these aggressive factors, and the most widely used type of prescribed and consumed pharmaceuticals, are non-steroidal anti-inflammatory drugs (NSAIDs).

The high use of NSAIDs is due to their many beneficial effects, including their antipyretic, analgesic, and anti-inflammatory activities. However, they are also highly aggressive towards the gastric mucosa, such that gastric damage is the most common adverse effect of their use. The biochemical basis of these lesions is due to the inhibition of cyclooxygenase (COX), which decreases the production of prostaglandins, inhibits blood flow, and, consequently, the production of mucus and bicarbonate that protect the mucosa, as well as reducing the vasodilation necessary to repair these lesions [3,4].

Gastric damage can also be caused and promoted by an increase in the amount of reactive oxygen species (ROS). Homeostatic imbalance of the protective barrier of the gastric mucosa induces oxidative stress associated with gastrointestinal inflammation, further damaging the epithelium, and resulting in a dysregulation of antioxidant enzyme activity. Lipid peroxidation plays an important role in the generation of ROS, where the end products of this reaction, such as malondialdehyde (MDA) and 4-hydroxynonenal (4-HNE), are indicators of ROS-mediated gastric damage [5]. The body suffers oxidative stress when oxidants increase or antioxidants are insufficient; consequently, cellular metabolism is affected. These factors are considered the pathophysiological basis of gastric injury exerted by this group of drugs. Moreover, improper use by the patient can exacerbate adverse effects which damage blood vessel walls and, depending on the depth of the injury, the damage may be severe. This explains a considerable number of variables studied, such as edema, petechial stippling, hyperemia, and, in extreme cases, hemorrhage [6].

Omeprazole (OMP) is simultaneously prescribed with NSAIDs to mitigate their gastric side effects. It is a proton pump inhibitor that lowers the production of gastric acid, and is also commonly used in the treatment of peptic ulcers, according to its ability to alkalinize the gastric mucosa and exert mucosal protection. However, there are also side effects to its use, such as potential deficiencies of vitamin B_12_, calcium, and magnesium, and possible association with dementia, gastric cancer, and chronic kidney failure [7]. Thus, the common side effects of NSAIDs and the drugs used to treat them make it a necessity to look for alternatives in order to reduce and/or prevent gastric injury.

One strategy to exert protective effects on the gastric mucosa is the antioxidant activity, both endogenous [8] and exogenous [9], exhibited by some molecules. Exogenous antioxidants, particularly dietary ones, can act as gastroprotective agents by inhibiting lipid peroxidation and free radical generation, decreasing gastric acidity, increasing antioxidant activity, and regenerating the gastric mucosa. There are several studies evaluating the gastroprotective capacity of different food matrices and extracts by inducing gastric damage in in vivo assays using murine models [10,11,12,13].

In recent years, quinoa (*Chenopodium quinoa* Willd.) has become very popular in human nutrition, particularly due to its high nutritional value and content of bioactive compounds [14,15]. For example, studies have shown that it has a high biological activity [16,17], where some phenolic acids and flavonoids, such as kaempferol and quercetin glycosides, predominate [18]. Quercetin in particular is a flavonoid with high antioxidant potential, from which various effects are derived, such as the inhibition of lipid peroxidation [19,20], antihistamine properties, increasing mucus production, and decreasing gastric secretion and acidity [21], among others.

Interest in preparing food products with pseudocereals from quinoa and other Andean grains has increased due to the various attributes of their bioactive compounds, which have a high antioxidant potential with the application of various heat treatments [22,23,24,25]. Clinical and experimental studies have shown that the consumption of pseudocereal grains has been inversely associated with the development of chronic non-communicable diseases, where antioxidant activity has been one of the most studied mechanisms to which its numerous protective effects have been attributed [26,27,28,29,30,31,32,33]. On the other hand, as far as we know, there is no information about the activity of a functional food formulated from quinoa flour on gastric damage.

According to the aforementioned information, the aim of this work was to evaluate the gastroprotective activity of quinoa cookies on ibuprofen (IBP)-induced gastric ulcers in a murine model. The effect of cookies was compared with omeprazole (OMP) as a reference drug, due to its capacity to prevent gastric damage through the activation and degradation of luminal gastric acid.

## 2. Materials and Methods

### 2.1. Formulation and Preparation of Quinoa Cookies

White quinoa was obtained from a local market in the city of Hermosillo, Sonora, Mexico (Costco Wholesale, Hermosillo, Mexico); Quinoa grain was baked in a conventional microwave oven (LG Electronic Appliances, model MS2047GR, Tianjin, China with a maximum power of 1650 W, equivalent to 100% power) for 14 min. The sample was then rapidly cooled in an ice bath, and then placed on aluminum trays to dry at a temperature of 45−55 °C. The dry sample was ground (Laboratory Mill) to a particle size of <0.5 mm and stored at −20 °C [34].

The flour obtained was used to prepare cookies following the procedure described by the AACC, 2000 [35] (method 10–50D), with some modifications. The quinoa cookie (MQC) formulation consisted of the following ingredients: 2.5 g of baking soda, 2.5 g of salt, 50 g of butter, 40 g of sugar, 20 mL of water, and 100 g of microwave-treated quinoa flour. Preparation consisted of beating the butter and sugar to create a paste, then adding the powdered ingredients; the flour, baking soda, and salt were mixed for 2 min until a homogeneous mixture was created. The dough was rolled out with a rolling pin to a thickness of 6 mm, and a 25 mm diameter cookie cutter was used to shape the cookies. The cookies were placed on a 20 × 30 cm baking tray, baked at 160 °C for 10 min, cooled, ground, and stored at −20 °C until analysis. Cookies formulated with raw quinoa (with no thermal procedure applied to it) were prepared in the same way and labeled as RQCs.

### 2.2. Determination of Total Phenolics, Flavonoids, and Antioxidant Capacity

A methanolic extraction was first carried out on the MQC and RQC samples as described by Salazar Lopez et al. (2016) [36]. Determination of total phenolic content (TPC) was performed as reported by Ruiz-Hernández et al. (2022) [37]. Total flavonoid content (TFC) was performed with the aluminum chloride method as described by Valenzuela-González et al. (2022) [25]. Antioxidant capacity was measured by ABTS assay as described by Ruiz-Hernández et al. (2022) [37].

### 2.3. In Vitro Gastrointestinal Digestion

In order to estimate the bioaccessibility of TPC, TFC, and antioxidant activity, in vitro gastrointestinal digestion conditions were simulated in two phases (gastric and intestinal). As described by Salazar López et al. (2018) [38] with slight modifications. For the gastric phase, 1 g of MQC and RQC was weighed, to which 5 mL of 0.2 M HCl-KCl buffer solution were added, and the pH was adjusted to 1.5. Next, 667 μL of pepsin solution (300 mg/mL) was added, and the tubes were incubated for 1 h under continuous shaking in a water bath at 37 °C (Precision Scientific Mod. 66800, Winchester VA, USA). Three tubes were then removed from each sample and placed in an ice bath, and the remaining samples underwent intestinal digestion. An amount of 9 mL of phosphate solution (0.1 M and pH 7.5) was added to each gastric digestion tube, and the pH was adjusted to 7.5. Then, 1 mL of pancreatin solution (17 mg/mL) and bile salts (80 mg) was added. The mixture was incubated for 6 h under the same conditions to obtain the intestinal digest. Each digestion phase included a reagent blank, and the intestinal samples were centrifuged for 10 min, at 1500× *g* and at 4 °C. The recovered supernatants were redissolved in 50% methanol, filtered (Econofltr Nyln 0.25 mm and 0.45 μm, Santa Clara, CA, USA), and stored at −20 °C in amber vials until further analysis.

Equation (1) was used to evaluate the effect of gastrointestinal digestion on the bioaccessibility of TPC, TFC, and antioxidant activity, as follows:

(1) $$B\left(\%\right)=\frac{A}{B}\times 100$$

where A is TPC, TFC, or antioxidant activity quantified in each sample after the gastric and intestinal digestion phases, and B represents the same variables in the samples before the in vitro gastrointestinal digestion.

### 2.4. Gastroprotective Activity

#### 2.4.1. Animals and Experimental Diets

All experimental procedures involving living organisms were previously evaluated and approved by the Internal Committee for the Care and Use of Laboratory Animals (ICCULA) of the University of Sonora (PR-13/15/2024, 5 April 2024). Experiments were carried out in accordance to the International Guidelines for the Care and Use of Laboratory Animals, as well as applicable national and international regulations.

A total of 38 Wistar albino rats weighing 180–200 g were used; 8 were used for a preliminary experiment to determine the appropriate IBP induction time, and 30 (n = 6/group) for the experiment reported. The animals were obtained from the Animal Experimentation Laboratory of the Department of Food Research and Graduate Studies (DIPA) of the University of Sonora. They were first acclimated for one week, with a 12:12 h light/dark photocycle and a temperature of 20–25 °C, relative humidity of 40–70%, and free access to food and water.

The experiment lasted a total of 30 days, during which the experimental diets were administered. Animal weight was measured once a week, and a welfare index was performed using the Rat Grimace Scale (RGS) [39]. A semi-purified diet based on the AIN-76A was used and referred to as basal diet [40], with modifications on two of the five dietary groups: Group 4: 20% of microwave quinoa cookies and Group 5: 0.20% of quercetin, a safe dosage [41], as shown in Table 1.

#### 2.4.2. Nonsteroidal Anti-Inflammatory Drug (NSAID)-Induced Gastric Damage

A 30-day trial was conducted to evaluate the preventive gastroprotective effect of quinoa cookies. Five groups of six animals were randomly prepared; Group 1 received the basal diet (BD) (control), Groups 2 and 3 received BD; Group 4 received BD + 20% microwaved quinoa cookie (MQC); Group 5 received BD + 0.20% quercetin (Q). At the end of the experiment, the animals were subjected to an 8 h fasting period and intragastrically administered the follow drugs: Group 1 (control) (2 mL of saline solution); Groups 2, 3, 4, and 5, 100 mg/kg bw of IBP. Group 3 was also administered 20 mg/kg bw of OMP 1 h before IBP.

IBP was chosen as a representative NSAID due to it being a relatively mild compound, since our intention was not to cause extreme gastric damage. A very high dose or other aggressors are sometimes used to maximize lesion number and/or severity, which was not the purpose of the present work.

### 2.5. Macroscopic and Microscopic Analysis

Two hours after the treatment with IBP, the animals were intraperitoneally anesthetized with pentobarbital sodium (0.2 mL/kg bw), blood was drawn by cardiac puncture, and the animals were euthanized by cervical dislocation. The stomachs were removed, opened along the greater curvature, gently rinsed with cold saline solution to remove the gastric contents, and blood clots were examined macroscopically for mucosal lesions. The insides of the stomach were photographed with an iPhone 15-iOS16 camera for the determination of gastric damage index (GDI).

GDI scores were calculated using a semiquantitative scale, following the methodology of Vendramini-Costa et al., 2014 [42], which is based on the number of lesions in the gastric mucosa; Table 2 shows the scores used for this scale, which were used to calculate the percentage of gastric damage index (GDI%), as shown in Equation (2):

(2) $$GDI\%=\frac{{GDI}_{i}-{GDI}_{t}}{{GDI}_{i}}\times 100$$

where GDI_i_ is the number of GDI points of the control group, and GDI_t_ is the number of GDI points of the different experimental groups.

For histopathological analysis, the stomach samples were preserved in 10% formaldehyde. The tissue was dehydrated using an ascending alcohol series (70, 90, 96, and 100%), cleared with xylol, and incubated in paraffin. Sections of 5 µm thickness were prepared using a microtome, placed in a cassette holder, and stained with hematoxylin and eosin (H&E) for subsequent analysis by light microscopy at 10x magnification.

Histological changes were scored as follows, in accordance to the method reported by Yang et al., 2017 [43]: (1) congested blood vessels (score: 0–4), (2) hemorrhage (score: 0–4), (3) inflammatory cell infiltration (score: 0–4), and (4) edema (score: 0–4).

### 2.6. Statistical Analysis

Data normality was determined using an Anderson–Darling test; an analysis of variance (ANOVA) and Tukey–Kramer’s test was then used to determine differences between means, with a level of significance of *p* < 0.05. Data analysis was performed in the statistical package JMP 5.0.1 (SAS Institute, Cary, NC, USA).

## 3. Results

### 3.1. In Vitro Gastrointestinal and Bioaccessibility of Total Phenolic Content (TPC), Total Flavonoid Content (TFC), and Antioxidant Capacity

**Figure 1:**
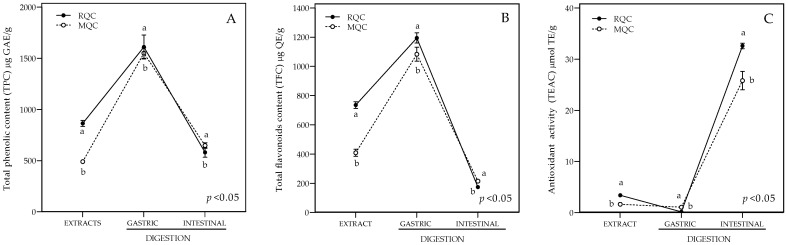
Results of in vitro gastrointestinal digestion of raw quinoa cookie (RQC) and microwaved quinoa cookie (MQC) on total phenolic content (TPC) (**A**), total flavonoid content (TFC) (**B**), and antioxidant capacity. Trolox equivalents antioxidant capacity (TEAC) (**C**). gallic acid equivalents (GAEs), quercetin equivalents (QEs), Trolox equivalents (TEs). Data are expressed as the mean ± SE. Different lowercase letters indicate significant differences (*p* < 0.05) (Tukey’s test).

Results of the TPC, TFC, and antioxidant capacity are shown in Figure 1. Before gastrointestinal digestion, the RQC samples showed higher values of TPC (864.4 ± 28.4 µg GAE/g), TFC (735.3 ± 23.0 µg QE/g), and antioxidant activity (3.4 ± 0.1 µmol TE/g) as compared to the MQC samples (490.7 ± 10.7 µg GAE/g; 408.7 ± 12.9 µg QE/g and 1.7 ± 0.1 µmol TE/g, respectively). After the gastric phase, both the RQC and MQC samples increased their values of TPC (1610.8 ± 117.3 vs. 1551.0 ± 51.1 µg GAE/g) and TFC (1194.4 ± 36.0 vs. 1083.4 ± 47.9 µg QE/g), with the RQC sample having higher values than the MQC sample. After the intestinal phase, the values of TPC and TFC were notably decreased for the RQC and MQC samples (580.6 ± 47.7 vs. 647.9 ± 27.0 µg GAE/g for TPC and 173.5 ± 5.1 vs. 214.7 ± 6.8 µg QE/g for TFC); similar values were obtained before digestion. It is relevant to highlight that, in the intestinal phase, the MQC samples had higher values of TPC and TFC as compared with the RQC samples (*p* < 0.05). Antioxidant activity was considerably higher after the intestinal phase as compared to the gastric phase and before digestion. The RQC samples showed higher antioxidant activity than the MQC samples after this same phase.

The bioaccessibility percentage was calculated considering data before the digestion of the RQCs and MQCs as 100% for TFC, TFC, and antioxidant activity. The results show that the MQC had higher bioaccessibility values than the RQC for both gastric and intestinal digestion, since it had the highest percentage of bioaccessibility for TPC (316%) and TFC (265%), while the RQC exceeded 100% bioaccessibility for total phenols (186%) and flavonoids (162%) in the gastric phase. Regarding the antioxidant activity, the intestinal phase showed the highest bioaccessibility, with 1564% MQC and 958% RQC.

### 3.2. Toxicity of Experimental Diets

The animals were observed continuously for the first 4 h after diet administration, and once a day for 30 d, with all observations being recorded systematically and individually for each animal. Additionally, following the Rat Grimace Scale proposal by NC3Rs [39], changes in the orbital tightening, nose/cheek flattening, ear changes, and whisker changes were documented. The results showed that the administration of experimental diets did not result in substantial changes in the rats that could indicate stress or harm. Additionally, no mortality was recorded; therefore, quinoa cookies and quercetin were classified as natural products with no apparent indicators of toxicity.

**Figure 2:**
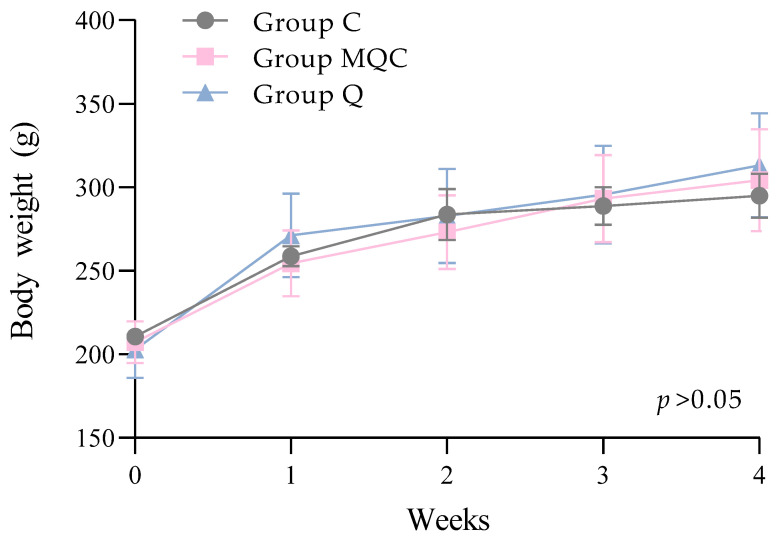
Body weight of the animals in the control, MQC, and Q groups. Data are expressed as the mean ± SE. *p* < 0.05 (Tukey test) compared to the control group.

The body weight of each animal was recorded weekly for all of the experimental diets. The changes in the body weight of individual animals were compared with those of the control animals. No significant changes were observed throughout the observation period in either male or female rats (Figure 2).

### 3.3. Assessment of Gastric Damage After Treatment with the MQC and Q

**Figure 3:**
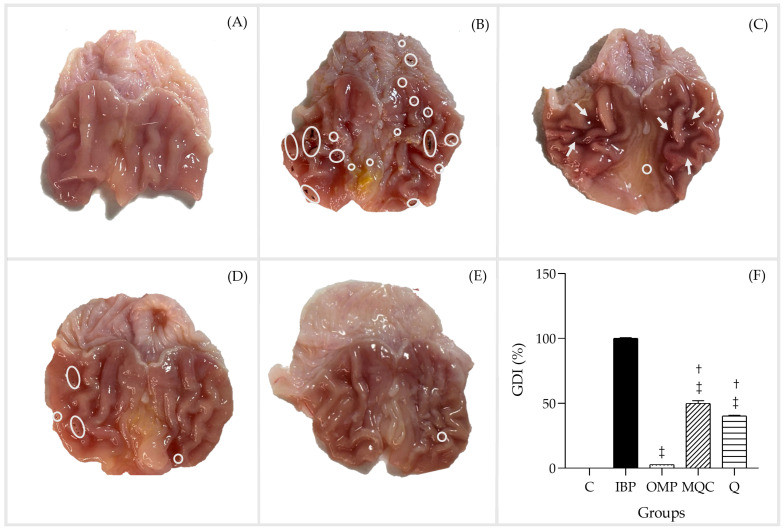
Effect of different treatments on macroscopic features of ibuprofen (IBP)-induced gastric injury in the stomachs of the experimental groups. Data are expressed as the mean ± SE. One-way analysis of variance was followed by the Tukey–Kramer post hoc test. Significance is represented as (‡) *p* < 0.05 as compared to the IBP group; (†) *p* < 0.05 as compared to the omeprazole group. (**A**) Control: basal diet (C), (**B**) ibuprofen (IBP), (**C**) omeprazole (OMP), (**D**) 20% microwave quinoa cookie (MQC), (**E**) 0.20% quercetin (Q), and (**F**) gastric damage index (%). Circles on figure indicate petechiae, and the arrows indicate bleeding/hemorrhage.

Figure 3 shows the macroscopic appearance of stomachs after 30 d of feeding the animals the basal and experimental diets, and treating them with IBP and OMP. Those in the control group were treated with only the vehicle (saline solution), and showed no gastric lesions, abnormal discoloration, or petechiae (Figure 3A), while Group 2 (IBP), which were treated with a single dose of IBP (100 mg/kg bw), developed acute gastric injury after 2 h of administration, according to multiple petechial puncta (<1 mm) (circles) (Figure 3B). As compared with the IBP group, gastric mucosal injuries showed a significant decrease in the injuries when Groups 3, 4, and 5 were administered OMP, MQC, and Q, respectively (Figure 3C–E). Notably, MQC 20% and Q 0.20% exerted significant decreases in gastric damage, which was 50% and 40.27%, respectively, although the OMP group had the least damage (3% *p* < 0.05) (Figure 3F). This group showed near-normal features with mild bleeding (arrows) and minor petechial lesions (>1 mm) (circles).

### 3.4. Effect of Different Dietary Treatments on Histological Features

**Figure 4:**
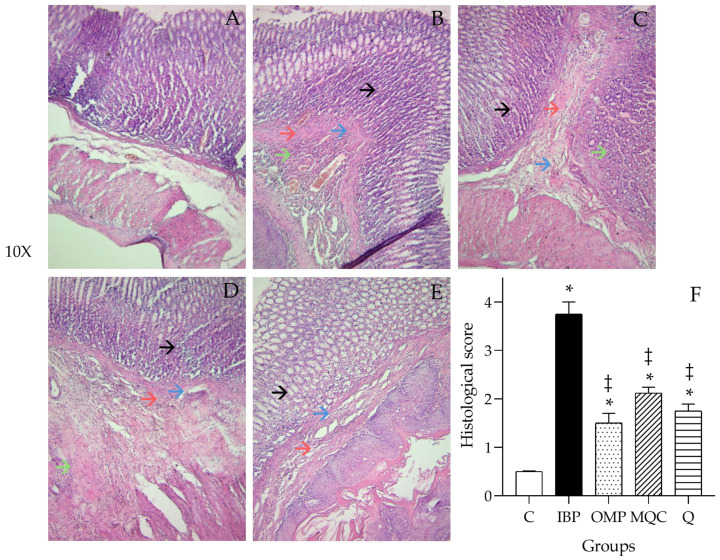
Effect of different treatments on ibuprofen (IBP)-induced gastric damage stained with hematoxylin and eosin (H&E, 10× light microscopy magnification) of the experimental groups. Data are expressed as the mean ± SE. Significance is represented as (*) *p* < 0.05 as compared to the control group; (‡) *p* < 0.05 as compared to the IBP group. One-way analysis of variance was followed by the Tukey–Kramer post hoc test. (**A**) Basal diet control (C), (**B**) ibuprofen (IBP), (**C**) omeprazole (OMP), (**D**) 20% microwave quinoa cookie (MQC), and (**E**) 0.20% standard quercetin (Q). (**F**) Histological score of gastric mucosal injury. Damaged gastric epithelium (→ black arrow), submucosa hemorrhage (→ green arrow), congested blood vessels (→ red arrow), and neutrophil infiltration (→ blue arrow).

Figure 4 shows histological analyses of gastric tissue using H&E staining. The results showed normal gland architecture in the control group; the submucosa was represented as a thin layer of connective tissue with multiple blood vessels in a normal state (Figure 4A). In IBP-induced gastric injury samples without pretreatment, congested blood vessels and neutrophil infiltration were observed in the gastric submucosa (Figure 4B). A thick layer of submucosa with congested blood vessels was observed in the OMP group (Figure 4C). The MQC group showed near-normal architecture of the gastric epithelium and pits, and less neutrophil infiltration (Figure 4D). The quercetin-treated (Q) group showed a similar architecture of the gastric epithelium and pits, similar to the MQC group (Figure 4E).

### 3.5. Antioxidant Enzyme Activities and Inflammatory Cytokine TNF-α

**Figure 5:**
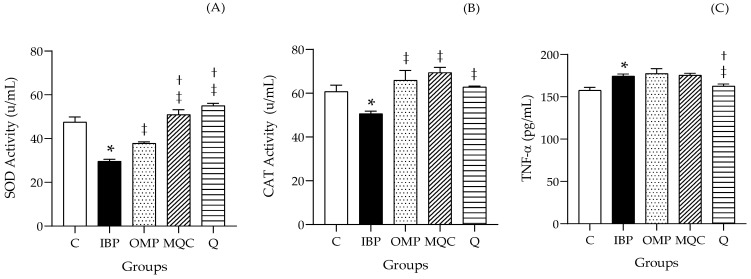
Effect of MQC and Q diets on endogenous antioxidant enzymes (SOD and CAT) and inflammatory cytokine TNF-α concentration on plasma of rats with IBP-induced gastric damage. Samples were analyzed in triplicate. (**A**) Superoxide dismutase (SOD) activity. (**B**) Catalase (CAT) activity. (**C**) Tumor Necrosis Factor Alpha (TNF-α). Control group (C), ibuprofen (IBP) group, omeprazole (OMP) group; 20% microwaved quinoa cookie (MQC); and 0.20% quercetin (Q). A one-way analysis of variance was performed with the Tukey–Kramer post hoc test. Significance is represented as (*) *p* < 0.05, as compared to the control group; (‡) *p* < 0.05, as compared to the IBP group; and (†) *p* < 0.05, as compared to the OMP group.

The results of the effect of enzymatic antioxidant activity on the plasma of rats fed different dietary treatments are shown in Figure 5. SOD (Figure 5A) and CAT (Figure 5B) activity was decreased in the IBP group as compared to the control group (*p* < 0.05). The MQC and Q groups showed an increase in SOD and CAT activities as compared with the IBP group, while the OMP group also showed an increase in enzymatic antioxidant activity as compared to the IBP group. Figure 5C shows the effect of different dietary treatments on the TNF-α concentration, where significant differences between the control group and the IBP group are apparent; thus, the generation of gastric damage in the model can be established. Of the treatments administered, only Group 5 (Q) showed a significant difference as compared to Group 2 (IBP).

### 3.6. Effect of Experimental Diets on Antioxidant Activity in Blood Plasma

Table 3 shows the antioxidant activity (ORAC) results obtained from the different treatments. The results showed a significant difference (*p* < 0.05) between the control and IBP group. The Q group had the highest antioxidant activity among the treatments, with no differences with the control group (C). The OMP and MQC groups did not show an increase as compared with the IBP group.

## 4. Discussion

Quinoa is a pseudocereal that is cultivated in South America, specifically, in the Andean region, although its cultivation has extended to other regions of the world in recent years [44]. Its nutritional value and biological potential have allowed it to be recognized as one of the most complete foods, with great benefits for human health [28]. Like most grains, it requires heat treatment prior to consumption, which has led to various studies that report a variation in its content of bioactive compounds, depending on the type of heat treatment applied [24,45,46]. Valenzuela González et al., 2023, applied microwave radiation to quinoa, obtaining flour with a higher content of phenolics and antioxidant activity as compared to an untreated quinoa flour [34]. The cookies formulated in the present study were prepared from microwave-heat-treated flour and analyzed regarding their total phenolic and flavonoid contents and antioxidant activity. RQCs showed higher TPC and TFC values and antioxidant activity as compared to MQCs. This is possibly attributed to the integrity of the RQC food matrix, which allowed these bioactive compounds to maintain a certain stability during the baking process, as compared to the MQC, which, when formulated with heat-treated quinoa flour, possibly made its bioactive compounds more susceptible to the baking process. It is essential to note that these results were obtained from the methanolic extracts of each cookie sample and may not accurately reflect the behavior of these compounds under gastrointestinal digestion conditions.

Gastrointestinal digestion is considered useful for estimating pre-absorptive events, such as the stability and bioaccessibility of phytonutrients from the food matrix [47]. In the present study, the cookies were subjected to a gastrointestinal simulation assay, which showed that the phenolic compounds quantified before digestion increased notably in the gastric phase (RQC > MQC). This could mean that the gastric digestion conditions significantly favored the release of phenolic compounds, which resulted in higher concentrations of TPC and TFC. These could have been covalently bound, conjugated (glycosides), or physically trapped in the food matrix. Similar results have been reported by Salazar López et al., 2018, in sorghum cooked by extrusion [38], potentially supporting this idea. In the same way, the RQC could have a higher release susceptibility of phenolic compounds as compared to the MQC. After the intestinal digestion process, the TPC and TFC values decreased considerably to values practically like those reported before digestion (RQC > MQC). The intestinal digestion conditions (digestion time, pH, and enzymes) undoubtedly contributed to this behavior by promoting the degradation of phenolic compounds, their re-polymerization, or their interaction with other components of the food matrix.

Up to this point, it is possible to assume that the RQC presented the best bioactive characteristics as compared to the MQC; however, it is important to highlight the concept of bioaccessibility, which is defined as the capacity of a bioactive compound to be released from the food matrix by the action of gastrointestinal conditions, and be available for absorption in the intestine [47]. Under this concept, it is relevant to highlight that the MQC had the highest percentages of TPC, TFC, and antioxidant activity as compared to the RQC. This reaffirms that the phenolic content in the food matrix does not always reflect its behavior under gastrointestinal digestion conditions. Furthermore, although the raw quinoa cookies displayed notable bioactive characteristics, it is important to highlight that their sensory properties were not acceptable, especially the flavor attribute, which was less appreciated by the panelists. This suggests that consumption of RQCs by actual human consumers is not likely to occur, since our choices of what foods to consume are based on their organoleptic properties, in addition to any potential bioactivities. This reinforces the fact that applying a microwave heat treatment to quinoa to obtain flour, and subsequently use it to prepare cookies, was a determining step in carrying out the in vivo study of gastroprotective activity.

NSAIDs are known for their anti-inflammatory action, which occurs by inhibiting COX enzymes; nevertheless, this mechanism of action is also responsible their potential to damage the gastric mucosa [48]. In spite of their beneficial effects, it is known that gastric ulcers may be associated with the use of NSAIDs, which underlines the relevance of countering this side effect.

IBP is the most widely consumed NSAID worldwide [49], and epidemiologic studies show that it is consistently ranked lower in toxicity among these kinds of drugs, while ketorolac ranks consistently high and causes damage to the gastric mucosa. However, NSAIDs in general are associated with some risk for upper gastrointestinal side effects, depending on frequency and doses [50]. It is for these reasons that IBP was selected over ethanol or another NSAID as a gastric damage inducer for the present work, aiming to induce minimal gastric damage, but which was as close as possible to real life use.

The chemical nature of NSAIDs allows them to interact with surface membranes and phospholipids [51]. This indicates that they may be absorbed and accumulate to concentrations that allow the uncoupling of mitochondrial oxidative phosphorylation, which promotes the generation of ROS, lipid peroxidation, neutrophil infiltration, apoptosis, and the inhibition of prostaglandin synthesis [42]. The generation of free radicals that cause oxidative stress is the main cause of damage to the gastric mucosa in response to an NSAID treatment. Triggering free radicals, like the hydroxyl radical or superoxide anion (OH and O_2_^−^), increases lipid peroxidation, damages the cell membrane, and depresses the endogenous antioxidant enzymes of the stomach [52].

The clinical relevance of anti-inflammatory drugs is unquestionable, and strategies to mitigate their side effects focus primarily on co-medication with other drugs, such as OMP, one of the most widely used drugs for this purpose. However, several side effects have been observed when it is administered chronically; thus, non-pharmacological alternatives are being heavily studied, especially directed at antioxidant gastroprotective therapies.

The gastroprotective activity of basal diets supplemented with quinoa cookies (20%) and quercetin (0.20%) was evidenced according to the results of the macroscopic (GDI), microscopic (histological score), and biochemical (antioxidant activity) analyses. The gastroprotective effects of quinoa’s bioactive compounds are supported by the results of various in vivo studies, highlighting their potential as a treatment and/or preventive agent for gastric lesions [10,11,53]. For example, Wang et al. (2023) demonstrated the gastroprotective effect of quinoa seeds, which was due to their antioxidant and anti-inflammatory effects [2].

Digestive cytoprotection decreases the size of gastric lesions in response to the ability of certain secondary metabolites to increase mucus synthesis, and its consequent ability to bind to the superficial gastric mucosa and exert its action as a protective layer, thereby eliminating the damage caused by the damaging agent (NSAID) [53]. OMP is a proton pump inhibitor with the ability to decrease the acid volume and, consequently, increase the gastric pH [54,55].

According to the macroscopic study, the untreated IBP group showed high punctuation in GDI as compared to the control group. When rats were given OMP (20 mg/kg) before the induction of gastric damage with IBP, a significant reduction in the GDI score was observed. The MQC and Q diets administered as a preventive treatment of IBP-induced gastric damage exerted a significantly lower GDI of 50% and 40.27%, respectively, as compared to IBP (100%). It was possible to observe that both OMP and the experimental diets showed a cytoprotective effect on the gastric mucosa, significantly delaying or inhibiting IBP-induced gastric damage. A microscopic analysis showed damaged gastric epithelium, submucosa hemorrhage, congested blood vessels, and neutrophil infiltration in the IBP group as compared with the control group. Since IBP is a nonselective NSAID, it follows a COX-dependent mechanism of action that includes a decrease in prostaglandin E2 (PGE2) biosynthesis. Such prostaglandin inhibition contributes to the pathogenic activity of NSAIDs, since PGE2 promotes multiple bioactivities that contribute to a net gastroprotective effect [56]. Gastroprotection agrees with the results observed after treatment with the OMP, MQC, and Q treatments, which significantly reduced the IBP-induced gastric damage. Although the present study did not specifically analyze PGE2, El Ashmawy et al. (2016) reports that one of the antiulcerative mechanisms of OMP is due to upregulating COX and PGE2; thus, MQC or Q could have a similar protective mechanism of action to OMP [57].

Mucosal blood flow is regulated by PGE2; decreased irrigation favors leucocyte and neutrophil infiltration. Under these conditions, inflammatory diseases could be developed and associated with the secretion of the enzyme myeloperoxidase (MPO). Nonselective NSAIDs like IBP can induce gastropathy dependent on neutrophil infiltration. Neutrophil infiltration catalyzes the production of proinflammatory and proapoptotic ROS [58,59].

Our results show that rats treated with MQC, Q, and OMP attenuated the decrease in blood flow, possibly by promoting PGE2 synthesis, which led to less evidence of neutrophil infiltration into gastric tissue. These results are supported by the histological analysis, where the MQC (20%) and Q (0.20%) groups showed a decrease in the inflammatory infiltration like the OMP group.

Interestingly, it is also possible that the increase in neutrophil infiltration could result in increased IBP-induced ROS formation. ROS produced by neutrophils promote lipid peroxidation in ulcers associated to IBP consumption due to an altered cellular membrane [60]. ROS may be neutralized by different enzymes, including SOD, glutathione peroxidase (GPx), and others. Free radicals can also be produced directly by IBP due to it inhibiting mitochondrial oxidative phosphorylation, which then induce a cellular imbalance in osmotic and lipid peroxidation, leading to tissue damage [59].

In the present study, the administration of IBP probably led to lipid peroxidation in gastric tissue, reflected in decreased SOD and CAT activity. An increase in the activity of both antioxidant enzymes was observed in rats treated with MQC and Q, suggesting its antioxidant properties. OMP showed a similar effect to diets supplemented with MQC and Q, also increasing the activity of said enzymes, although less noticeably. It is also known that OMP can scavenge different radicals [60], which contributes to its protective effect.

TNF-α is a proinflammatory cytokine that stimulates the acute phase of the inflammatory response and contributes to gastric injury. TNF-α is involved in the recruitment of neutrophils through the induction of adhesion molecules. TNF-α has the additional capacity to produce superoxide molecules by the accumulation and activation of neutrophils, finally leading to disturbances in microcirculation and the production of free radicals [59]. In this study, the proinflammatory cytokine TNF-α was increased in the IBP group as compared with the control group. Of the treatments administered, only the Q group showed decreased TNF-α levels as compared to IBP group. Several studies show that quercetin is the main flavonoid in quinoa, and exerts antioxidant activity capable of neutralizing free radicals, a bioactivity derived from its chemical structure that contains various hydroxyl groups responsible for hydrogen ion transfer. The antihistamine properties of quercetin have also been reported (histamine signaling promotes gastric acid production); thus, it can act as an antisecretory, increase mucus production, and reduce gastric secretion and acidity, thereby resulting in a net gastroprotective effect [21,61].

The present study reports that greater antioxidant activity was observed in the plasma of rats treated with quercetin (Q group) as compared to the IBP group. Various studies have suggested that antioxidants might effectively protect against gastric mucosal injury and inhibit the progression of gastric ulcers; thus, the good antioxidant capacity of the MQC may be responsible for this effect, according to its high phenolic and flavonoid contents. Phenolic compounds may donate hydrogen atoms from their hydroxyl groups [10,51,52]; the phenolics present in the MQC could exert their antioxidant activity directly or indirectly. For example, a direct mechanism may occur through capturing superoxide anions or other ROS species, which consequently inhibits their damage to the gastric tissue while, indirectly, they may stimulate the activity of SOD and CAT, which further neutralize free radicals.

Considering the results reported in the present study as a whole, it is possible to propose MQCs as part of a preventive dietary treatment for gastric damage as an alternative to the use of drugs like OMP, which, although they play an important role in the prevention of gastric damage, their adverse side effects on health are well documented [62]. The findings attributed to the MQC can be summarized as (1) a reduction in the macroscopic indicators of gastric damage; (2) the lesser modification of the architecture of the gastric tissue; (3) increased endogenous antioxidant enzyme activity (SOD and CAT) and the reduction in TNF-α in the quercetin group; and (4) increased antioxidant activity in the plasma of rats treated with quercetin.

Regarding quercetin, given its relevance as a gastroprotective agent, the results reported could mark the beginning of the exploration of other foods with significant contents of this flavonoid, and its possible contribution to the prevention and/or treatment of NSAID-induced gastric damage. These findings could lead to a first approach in studies on quinoa-based functional foods, while subsequent ones may strengthen them by, for example, evaluating lipid peroxidation indicators (such as MDA) and the levels of secondary metabolites that participate in the generation of gastric damage, like myeloperoxidase activity, prostaglandin synthesis, among others.

Finally, the authors would like to highlight some limitations of the present work. First, prolonged consumption of desserts, including cookies, may be nutritionally detrimental for carbohydrate (insulin resistance, high glycemic response, etc.) or lipid metabolism (changes to the serum lipid profile, obesity, etc.); the consumption of foods rich in phenolics could therefore be performed in a matrix that contains a lower sugar and fat content. Future studies could improve on this aspect of our experiment. Other food matrices could also be considered. Second, an animal model remains an approximation to human physiology which, although similar, does not fully encompass the complexity of factors like personal preference, a varied diet, other health issues, age, the consumption of other gastric irritants like alcohol, and multiple other variables unique to humans. Direct extrapolation to human beings should therefore be performed cautiously; human-derived data are therefore highly valuable and important for subsequent studies. Third, the nutritional effects of the cookies (or other food matrices used in future studies) could be considered; that is, the fat or protein content could have a particular effect not considered here. Fourth, the effect of the treatments regarding the effectiveness of IBP on analgesia was not addressed in the present work. Additional experiments are therefore required to determine if there are any changes on the biological activity of IBP on pain relief in the consumer.

## 5. Conclusions

The gastroprotective activity of quinoa cookies and quercetin included decreased gastric damage, the maintenance of gastric tissue architecture, the increase in endogenous antioxidant enzymes (SOD and CAT), and a decrease in the proinflammatory cytokine TNF-α. These findings are supported by the high intestinal bioaccessibility of total phenolics and flavonoids, and the antioxidant activity of the MQC. It was therefore determined that the in vivo data reported suggest a preventive gastroprotective effect on IBP-induced gastric injury. These results may offer a promising therapy for the treatment and/or prevention of NSAID-induced gastric injury, promoting the use of quinoa as a functional food in products with gastroprotective activity.

## Figures and Tables

**Table 1:** Experimental diet formulation for the five experimental groups (g/kg/d).

Ingredients	Group 1 C	Group 2 IBP ^1^	Group 3 OMP ^2^	Group 4 MQC	Group 5 Q
	g/kg/d				
Casein	17.90	17.90	17.90	17.90	17.90
Starch	36.28	36.28	36.28	21.53	36.13
Sucrose	7.14	7.14	7.14	7.14	7.14
Soybean Oil	3.57	3.57	3.57	3.57	3.57
Cellulose	5.71	5.71	5.71	5.71	5.71
Vitamin Mix	0.71	0.71	0.71	0.71	0.71
Mineral Mix	2.48	2.48	2.48	2.48	2.48
Microwaved Quinoa Cookie (MQC)	--	--	--	14.75	--
Quercetin (Q)	--	--	--	--	0.14
TOTAL (daily intake)	73.79	73.79	73.79	73.79	73.79

^1^ IBP was administered intragastrically at the end of the experiment to all groups except the control. ^2^ OMP was administered intragastrically at the end of the experiment to Group 3, 1 h before IBP. n = 6/group.

**Table 2:** Semiquantitative gastric lesion scale used to calculate gastric damage index (GDI).

Parameter	Points
Loss of normal morphology	1 point
Mucosal discoloration	1 point
Mucosal edema	1 point
Hemorrhages	1 point
Petechial points (up to 9)	2 points
Petechial points (>10)	3 points
Ulcers up to 1 mm	n × 2 points
Ulcers (>1 mm)	n × 3 points
Perforated ulcers	n × 4 points

**Table 3:** Plasma antioxidant activity (ORAC µg TE/mL) of rats treated with IBP, OMP, and experimental diets.

Groups	ORAC
C	7.21 ± 0.37 ^a^
IBP	5.71 ± 0.30 ^b^
OMP	5.36 ± 0.27 ^bc^
MQC	4.70 ± 0.16 ^c^
Q	7.40 ± 0.23 ^a^

Data are expressed as the mean ± SE. Different lowercase letters between columns are significantly different at *p* < 0.05 (Tukey’s test). Control group (C), ibuprofen (IBP) group, omeprazole (OMP) group; 20% microwaved quinoa cookie (MQC); and 0.20% quercetin (Q).

## Data Availability

The original contributions presented in this study are included in the article. Further inquiries can be directed to the corresponding author.

## Abbreviations

The following abbreviations are used in this manuscript:

•OH	hydroxyl radical
4-HNE	4-hydroxynonenal
BD	basal diet
C	control
CAT	catalase
COX	cyclooxygenase
GDI	gastric damage index
GPx	glutathione peroxidase
H&E	hematoxylin and eosin
IBP	ibuprofen
MDA	malondialdehyde
MPO	myeloperoxidase
MQC	microwave quinoa cookie
NSAIDs	nonsteroidal anti-inflammatory drugs
O2.-	superoxide radical
OMP	omeprazole
ORAC	oxygen radical absorbance capacity
PGE	prostaglandin
Q	quercetin
ROS	reactive oxygen species
SE	standard error
SOD	superoxide dismutase
TNF-α	tumor necrosis factor alpha
